# Managing diabetes in diabetic patients with COVID: where do we start from?

**DOI:** 10.1007/s00592-021-01739-1

**Published:** 2021-06-25

**Authors:** Angelo Avogaro, Benedetta Bonora, Gian Paolo Fadini

**Affiliations:** Department of Medicine, Unit of Metabolic Disease, University of Padova, Via Giustiniani 2, 35128 Padova, Italy

**Keywords:** SARS-CoV-2, COVID-19, Diabetes mellitus, Type 2, Metformin, Diabetes treatment

## Abstract

**Aims:**

COVID-19 has and still is sweeping away the national health systems worldwide. In this review, we sought to determine the evidence base proofs on the antidiabetic treatment capable to reduce the risk of COVID-19-related mortality.

**Methods:**

We have performed a systematic search of published articles using PubMed, and EMBASE from March 2020 to March 31st, 2021. We excluded editorials, commentary, letters to the editor, reviews, and studies that did not have mortality as an outcome. For metformin and insulin only, we performed a meta-analysis using Cochrane RevMan 5.2.

**Results:**

Among antidiabetic drugs, metformin was the only drug associated with a reduced risk of mortality. Conversely, insulin appears associated with an increased risk. The other classes of drugs were neutral.

**Conclusions:**

The totality of articles reports retrospective data strongly affected by “channeling bias” so that most of the existing results on each class of drugs are driven by the phenotype of patients likely to receive that specific drug by prescription.

## Introduction

A novel coronavirus was identified at the end of 2019: this virus is a positive-stranded RNA virus, which causes a severe acute respiratory syndrome coronavirus 2 (SARS-CoV-2). It can also generate a systemic disease defined as COVID-19 [1]. Diabetes mellitus (DM) plays an essential role in determining disease severity, particularly in patients with a previously unknown condition [2]. The tight control of blood glucose levels might be crucial to prevent severe courses of COVID-19: we found statistically significant correlations between glucose levels at admission in hospital and severity of disease progression [3]. Besides diabetes, obesity is an essential determinant of the severe course of the disease: obesity shifts severe COVID-19 severity to a younger age, i.e., young obese subjects are more likely to be admitted to intensive care units [4]. Central to the severity of the disease is whether hyperglycemia/diabetes modulates the antibody response to the virus, an issue still a matter of discussion. Some studies reported normal plasma immunoglobulin levels in the presence of diabetes, while in other studies, reduced levels of IgG and IgM have been reported [5, 6]. Unfortunately, the humoral response's complexity against SARS-CoV-2 in patients with hyperglycemia has not yet been entirely unveiled. Coagulation and aggregations defects' background existence further contributes to cardiovascular morbidity and long-term sequelae from COVID-19 infection [7]. Notably, patients with more severe illnesses, such as those with diabetes, might experience long-term damage. The lasting effects of COVID-19 are now called “long COVID,” a distinct syndrome, probably determined by a dysfunctional immune-inflammatory response, affecting even people who were never hospitalized for COVID-19 [8].

Some reports have described late sequelae involving cardiovascular, pulmonary, and neurological manifestations. There is limited information about the disease's underlying pathophysiology and its fallout on long-term prognosis, particularly in patients with diabetes and obesity, either with or without complications. In light of these reasons, diabetes imposes a tremendous additional burden to the COVID-19 pandemic, before, during, and after the infection [9].

To complicate this scenario further, COVID-19 may cause diabetes by inducing hyper-inflammation, directly infecting pancreatic endocrine cells, leading to impaired insulin secretion [10].

A patient with diabetes must face several challenges when infected by SARS-CoV-2: the lack of exercise, which leads to an increase in body weight, mental stress, the lack of vitamin D, and above all, the need to keep adequate glycemic control [11]. All these variables pertain to patients who have stable glucose control and can self-manage the disease. Instead, a remarkable proportion of patients are old, with comorbidities and long-term complications: those who need to refer more frequently to healthcare facilities because of inability to use virtual visit technologies [12]. Several therapeutic approaches have been proposed for patients with type 2 diabetes in this complicated context, but very few are supported by robust evidence. This narrative review's scope highlights the proof we have so far to manage diabetes in the context of the COVID-19 pandemic.

## Metformin

Metformin, the first-line treatment for patients with type 2 diabetes, possesses several vital effects on the immune system, the most important being the reduced expression of anti-inflammatory cytokines, the stimulation of antigen-specific T cell responses, the reduction of infiltration of monocytes or macrophages into diseased tissues, the reduction of number and inhibition of the function of neutrophils [13]. Therefore, metformin could be considered a drug of choice for the treatment of diabetes in patients affected by COVID-19 [14]. Furthermore, metformin decreases the concentration of neutrophil extracellular traps (NETs), a neutrophil cell death program, increased in patients with type 2 diabetes, instrumental to anti-microbial defenses, and involved in tissue damage [15] even during COVID-19 [16, 17].

Lukito and colleagues showed in a meta-analysis including nine studies with 10,233 subjects, that metformin is associated with lower mortality both in non-adjusted (OR 0.45 [0.25, 0.81], *p* = 0.008) and adjusted model (OR 0.64 [0.43, 0.97], *p* = 0.035) [18]. To estimate potential “channeling bias,” we performed a meta-analysis of studies with and without propensity score matching in which the role of metformin on COVID-19 mortality was assessed. As for all the other antidiabetic drugs, the data on the effect of metformin treatment on COVID-19 severity and mortality (Table 1) available from the literature are retrospective, frequently obtained in a small number of patients without adequate propensity score matching or robust assessment of residual bias. This is important since metformin could be a potential marker of shorter diabetes duration or absence of comorbidities such as chronic kidney disease, an independent predictor of mortality in patients with COVID-19. Also, not using metformin as the first line, or as concomitant treatment in more advanced lines of therapy may be a proxy of inappropriate diabetes care delivery, which would per se drive bad outcomes.

**Table 1:** Studies in which association between DPP-4 inhibitor treatment for diabetes and COVID-19 mortality was assessed

Author	Patients	Setting	Type of study	Mortality
Fadini et al. [33]	403 hospitalized (85 with diabetes, 9 on DPP-4i))	In- and outpatients	Retrospective	Similar disease outcome between DPP-4i users and non-users
Silverii et al. [25]	159 (13 on DPP-4i)	Epidemiological observatory	Retrospective	1.0 (0.51–2.14)
Rhee et al. [34]	832 (263 on DPP-4i)	In- and outpatients	Retrospective	Prior use of a DPP-4i did not affect mortality or the clinical severity of the disease
Solerte et al. [35]	338 (169 on DPP-4i)	Hospitalized	Case–control retrospective	Sitagliptin at the time of hospitalization was associated with reduced mortality (18% vs. 37% of deceased patients; hazard ratio 0.44 [95% CI 0.29–0.66]; *P* = 0.0001)
Mirani et al. [36]	90 (11 on DPP-4i)	Hospitalized	Retrospective	Use of DPP-4i was significantly and independently associated with a lower risk of mortality (aHR 0.13, 95% CI 0.02–0.92; *P* = 0.042)
Pérez-Belmonte et al. [37]	1297 on glucose-lowering drugs in monotherapy and 465 in combination with metformin	Spanish society of internal medicine's registry of COVID-19 patients hospitalized	Retrospective	No at-home glucose-lowering drugs showed a significant association with in-hospital death;
Zhou et al. [38]	2563 (127 on DPP-4i)	Hospitalized	Retrospective	No significant association with mortality and adverse outcomes
Israelsen et al. [39]	GLP-1 RAs (*n* = 370) and DPP-4i (*n* = 284) compared with sodium-glucose cotransporter-2 inhibitors (SGLT-2i) (*n* = 342	Nationwide registry	Retrospective	No differences in hospital admission and severe outcomes in those on incretin treatment
Roussel et al. [40]	2449 DM (596 on DPP-4i)	Hospitalized	Retrospective	Day 7 (OR [95% CI]: 0.95 [0.77–1.17]) or Day 28 (OR [95% CI]: 0.96 [0.78–1.17])
Noh et al. [41]	586 DM (453 on DPP-4i)	Nationwide registry (health insurance review and assessment service database linked with the korea disease control and prevention agency database)	Retrospective	DPP-4i use was insignificantly associated with all-cause mortality (HR 0.74, 95% CI 0.43–1.26) and severe manifestations (HR 0.83, 95% CI 0.45–1.53)
Wargny et al. [26]	2796 DM (615 on DPP-4i)	Hospitalized	Retrospective	Predictor of discharge 1.22 (1.02, 1.47) Neutral on death 0.83 (0.65, 1.05)

To verify whether inadequate sensitivity analysis may account for a disproportion in the association between metformin and COVID-19 mortality, we screened the English literature for studies reporting SARS-CoV-2 infection and/or COVID-19-related mortality in metformin users. The search string “metformin AND COVID-19” was run in PubMed, Scopus, and Cochrane Library: the meta-analysis was performed, including all observational studies reporting mortality in patients with diabetes and COVID-19 and then splitting the analysis between studies with and without propensity score matching. All estimates were thus reported as risk ratios and 95% confidence interval (CI). The random-effect model was used to obtain pooled RR. Heterogeneity was assessed using the I^2^ test. Review Manager version 5.3 was used to perform the meta-analysis. Five studies were included in the analysis.

**Fig. 1:**
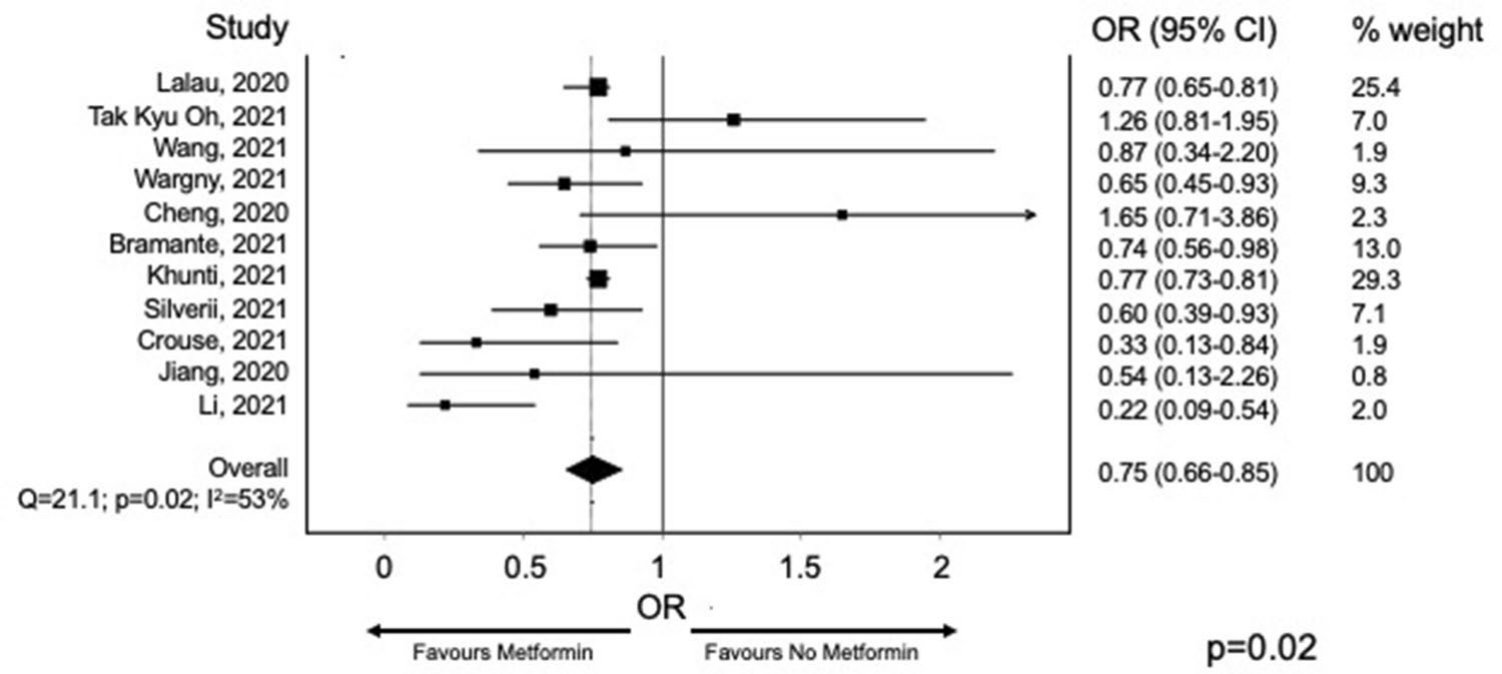
Risk for mortality for metformin users and other glucose-lowering medication users. Risk ratio with 95% confidence intervals

**Fig. 2:**
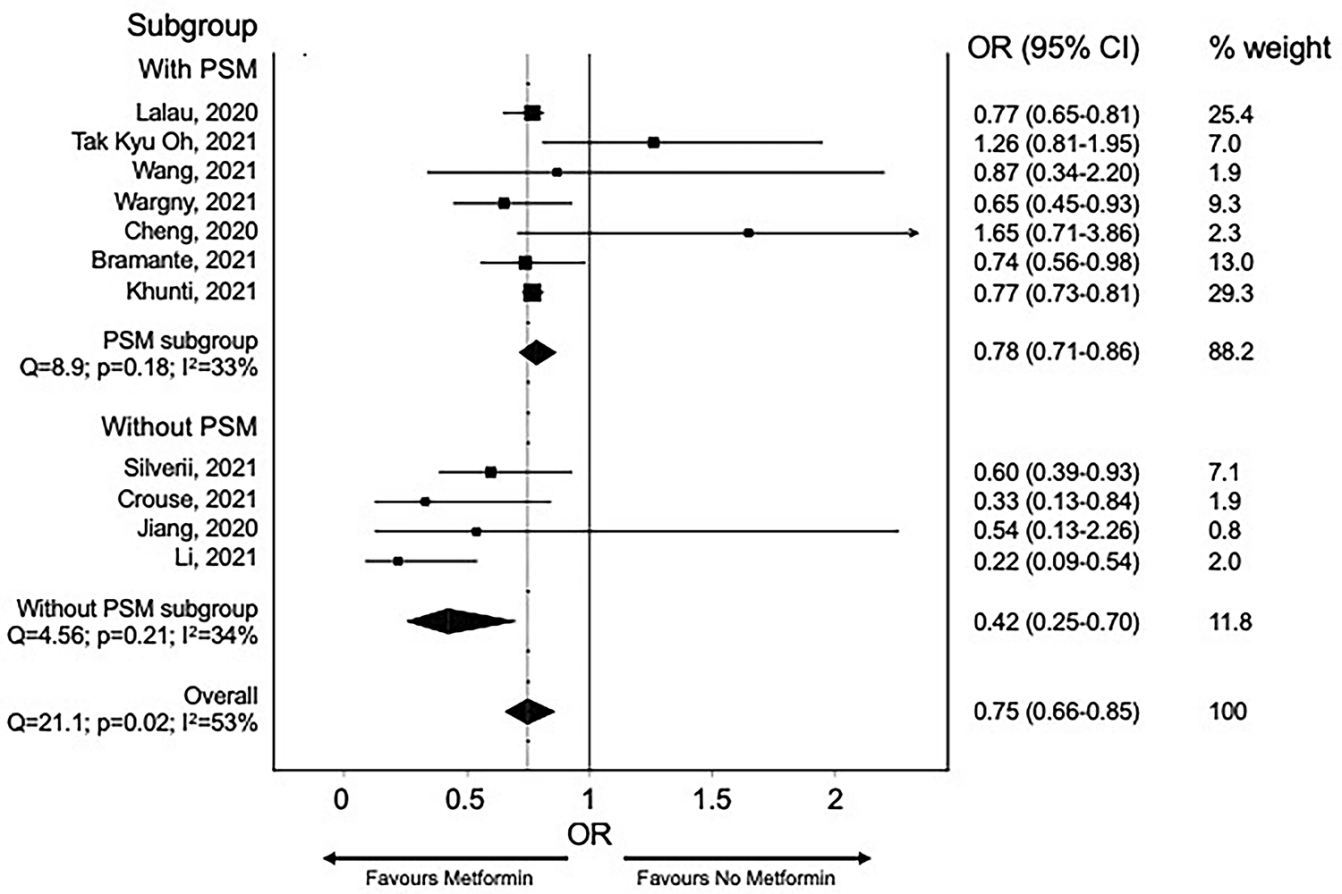
Risk for mortality for metformin users and other glucose-lowering medication users in studies with (top panel) and without (propensity score matching). Risk ratio with 95% confidence intervals

Despite a significantly lower heterogeneity between studies in which sensitivity analysis was performed, there is a comparable reduction in mortality risk in those treated with metformin with an overall risk reduction of 25% (Figs. 1 and 2). In their recent work, Khunti and colleagues, after an appropriate propensity score matching, show that the use of metformin was associated with a 23% decrease in the hazard ratio for COVID-19-related death [19]. The main concern during metformin treatment, especially in those with reduced kidney function and septic conditions, is lactic acidosis development. Cheng and coworkers showed that more individuals (*n* = 20; 2.95%) in the metformin group developed acidosis than the non-metformin group (*n* = 12;1.77%; *p* = XYZ) [20]. As expected, patients with acidosis in the metformin group took higher doses of the drug, had a worse kidney function, and a more severe COVID-19 disease than those who did not develop acidosis. Although metformin therapy before hospitalization does not appear to worsen the outcome of hospitalization, in patients with the long-COVID syndrome, a decreased renal function could be observed. Therefore, in patients with diabetes and persistent COVID-19 disease, glomerular filtration rate should be monitored to titrate metformin properly and withdraw it promptly below 30 ml/min/1.73 m^2^ [21].

## Sulphonylureas

The COVID-19 pandemic has hit the under-developed countries where the sulphonylureas (SUs) are the most prescribed drugs for diabetes. SUs acts mostly by stimulating glucose-independent insulin secretion by the beta cells where they close the potassium-ATP dependent channels thus favoring calcium influx and the release of the hormone. There is remarkably little information on the effect of SUs on COVID-19 severity and related mortality. However, there is no reason to believe, besides their ability to decrease plasma glucose, that they could potentially interfere with the host response to the virus. Singh Tomar and coworkers have identified a potential role of gliclazide in blocking the E protein an ion channel which could favor virus entry into cells [22]. A study in Korea reported neutrality for COVI-19 mortality (OR 0.84; 95% CI, 0.23–3.09) in SUs users vs. non-users [23]. A similar result was reported by Izzi-Engbeaya for deaths within 30 days (OR 0.66; 95% CI, 0.30–1.52) in univariate analysis [24] and by Silverii and colleagues [25]. The Coronavirus SARS-CoV-2 and Diabetes Outcomes (CORONADO) study also found a neutral effect of SUs/glinides on mortality (OR 0.83; 95% CI, 0.67–1.03) within 28 days [26]. In a nationwide observational cohort study done with data from the National Diabetes Audit for people with type 2 diabetes, Khunti and colleagues have reported that the use of SUs marginally but significantly reduced COVID-19 mortality whereas this was not observed for glinides [19]. Notwithstanding their possible neutrality on mortality, SUs should be used with extreme caution in patients with diabetes given their ability to provoke hypoglycemia. This is further aggravated in those with either impaired renal function or poor caloric intake. They should be withdrawn in the presence of severe/critical for of the disease.

## Pioglitazone

Several review and hypotheses papers on antidiabetic treatment during COVID-19 infection endorse the use of the peroxisome proliferator-activated receptor-γ agonists pioglitazone as an ideal drug. The reason for this benefit is based on the potential anti-inflammatory activity of this drug. Pioglitazone reduces the cellular adhesion molecules, modulates the lymphocyte T helper, suppresses the nuclear factor-kB and the inflammatory response, and the consequent expression of the pro-inflammatory cytokines [27]. Several studies have also shown that pioglitazone possesses the ability to decrease viral infectivity, at least in the context of viral hepatitis C [28]. The only research demonstrating the role of thiazolidinediones in the context of COVID-19 disease is from Khunti and colleagues who showed substantial neutrality in terms of mortality [19]. There are either no prospective or retrospective observational studies or randomized controlled studies showing the benefit of pioglitazone treatment on COVID-19 disease severity or mortality. Nonetheless, there is no reason to discontinue the drug in patients with either asymptomatic or moderate disease in patients with type 2 diabetes.

## Dipeptidyl peptidase 4 inhibitors (DPP-4i)

Fifty percent of glucagon-like peptide-1 (GLP-1) is degraded in roughly 1 min by the dipeptidyl peptidase 4 (DPP‐4) or CD26, a 110 kDa peptidase, a transmembrane glycoprotein almost ubiquitously expressed in the surface of many cells including epithelial and endothelial cells and immune cells. DPP-4 also exists as a soluble form in the circulation, where it retains its enzymatic activity [29]. DPP-4 promotes T cell activation and proliferation, modulates other immune cells' function, and stimulates pro-inflammatory cytokines. Therefore, DPP4 is potentially involved in the regulation of both innate and acquired immunity [30]. Human DPP-4 has been identified as a functional receptor for the spike glycoprotein of the Middle East Respiratory Syndrome coronavirus (MERS- CoV), which is phylogenetically correlated SARS-CoV-2 [31]. Thus, DPP4 inhibition might interfere with the entering of the virus into host cells.

However, there is no evidence that DPP4 is also a receptor for SARS-CoV-2. However, DPP-4 inhibition may antagonize SARS-CoV-2 virulence by reducing the cytokine storm and lung inflammation[32]. DPP4-i are widely used to treat type 2 diabetes: their purported role of inhibitors of viral entry into the cell has pushed several investigators to repurpose their use to treat COVID-19 infection, as shown in Table 1.

Our research found no significant difference in the rate of intensive care unit (ICU) admission or death between DPP-4i users and non-users [33, 42]. Furthermore, we observed that the rate of DPP-4i use was also similar in patients with diabetes mellitus hospitalized with COVID-19 pneumonia and with pneumonia of other etiology. At variance, in a case series involving 387 patients with COVID-19 admitted to hospital in Lombardy (Northern Italy), it has been shown that treatment with DPP-4i was associated with a lower mortality rate independently from confounders (adjusted HR 0.13; 95% CI 0.02–0.92)[36]. DPP-4i users needed non-invasive mechanical ventilation less frequently, suggesting less severe pneumonia. A less extreme course of the disease has been reported by Sang Youl Rhee and colleagues, who showed that DPP-4 users have a 54% less risk to have a severe clinical course than no-users[34]. It should be noted that this result was based on 11 patients only, who were treated with DPP4i. In another multicenter, case–control, retrospective, observational study conducted in Northern Italy hospitals, the use of sitagliptin was associated with reduced mortality (18% vs. 37%, *p* < 0.001) after multiple adjustments[35]. Notably, most of the studies were performed in a relatively small number of patients; therefore, the results were exposed to considerable bias. The CORONADO study, a nationwide multicenter observational study conducted in France, included 1317 patients hospitalized for SARS-CoV-2 with a history of DM or newly diagnosed DM, 21.6% of the participants were on DPP4i [43]. This study showed no association between a severe course of COVID-19 and a treatment with DPP4i before admission (OR 1.01; 95% CI 0.75–1.34). Other studies with a smaller number of patients confirmed these results. In a meta-analysis of 7 studies, we found an unbiased estimate of the risk ratio of COVID-19-related mortality among users of DPP4i (0.81; 95% CI 0.57–1.15) [42]. At variance, in their study, Khunti and colleagues have found that treatment with DPP-4i was associated with a small 1.07 (1.01–1.13) but significant increase in mortality [19]. The DPP-4i are preferentially prescribed to older and frail patients: thus, their findings would also confirm this class of drug a “channeling bias.”

Therefore, in the absence of prospective studies, the retrospective research available so far provides inconclusive results and conflicting evidence to consider DPP4i protecting against COVID-19.

## Glucagon-like peptide 1 receptor agonists (GLP-1RA)

GLP-1RA can affect immune response and inflammation: these compounds increase the number and activity of invariant natural killer T (iNKT) cells, a T cell population that exerts an essential role in recognizing both foreign and self lipid antigens [44]. GLP-1 secretion increases in response to cytokine surge [45] and bacterial debris [46]. GLP-1 acts locally to modulate intestinal inflammatory responses through the canonical GLP-1 receptor expressed on intestinal intraepithelial lymphocytes in the small and large bowel[47]. GLP-1RA possesses an anti-inflammatory activity in the human umbilical endothelial cells (HUVEC), where decrease intracellular ROS production by inhibiting the induction of gp91(phox) and p22(phox), a subunit of NADPH oxidase, by TNF-α [48]. In light of these experimental premises, GLP-1RA could be considered an ideal drug to counteract the pro-inflammatory cytokine response after severe COVID-19 infection. Five retrospective studies are available on the effect of GLP-1RA on COVID-19-related mortality [19, 25, 26, 39, 49]. None of these show that the use of GLP-1 before SARS-CoV-2 infection could potentially interfere with the disease's course. However, since this class of drugs not only to normalize HbA1c but also to decrease inflammation and restrain the pro-inflammatory response, it would be interesting to assess their role in prospective studies.

## Sodium-glucose cotransporter 2 inhibitors (SGLT2i)

SGLT2i rapidly inhibits renal glucose reabsorption by 30–50%, reducing blood glucose, body weight, and blood pressure. Cardiovascular outcome trials, both in patients with and without diabetes, have provided evidence that SGLT2i treatment is associated with remarkably positive cardiovascular outcomes [50]. Interestingly, both experimental and human data indicate that SGLT2i blunts inflammation with a specific protective effect exerted in the kidney and the liver [51]: these drugs may reverse molecular processes related to inflammation by decreasing interleukin-1, interleukin-6, and tumor necrosis factor 1 receptor. SGLT2i modify macrophage polarization from proinflammatory M1 to anti-inflammatory M2 [52]. The available studies on SGLT2i and COVID-19-related mortality report conflicting results. Sainsbury and colleagues did not assess mortality but reported neutrality in terms of susceptibility to SARS-CVoV-2 infection [53]. Silverii in a small group of patients reported neutrality toward COVID-19 mortality [25], while Khunti and colleagues showed that treatment with SGLT2i was associated with 18% reduction in mortality, despite a significantly higher HbA1c in those on this treatment [19]. SGLT2i increase urinary glucose and Na + excretion, urine volume, and solute-free water reabsorption (TcH2O): therefore, their potential to induce dehydration and increase ketogenesis, abstain from their use during severe COVID-19 infection. Vitale and colleagues have reported a cluster of euglycemic DKA cases in patients with type 2 diabetes mellitus using SGLT2is who developed SARS-CoV-2 infection [54]. In the light of these findings, their use is probably restricted to patients with either asymptomatic or mild clinical course of COVID-19. Those who are already on SGLT2i should receive an adequate amount of liquids matching the urinary loss induced by these drugs to a. avoid dehydration b. avoid over-hydration, especially in patients with heart failure (HF). In HF patients is essential to titrate loop diuretics since SGLT2i could potentiate their effect. Finally, patients with COVID-19 infection should be warned to ingest at least 50% of total calories as carbohydrates to avoid DKA.

## Insulin

Insulin treatment is required when patients with diabetes (including newly-detected) and COVID-19 have either a severe or critical clinical course of the disease. This approach is dictated by the fact that insulin is a physiological hormone with no adverse effects other than hypoglycemia; it can be easily titrated when given intravenously to maintain a target plasma glucose level. For non-critically ill patients, the American association of clinical endocrinologists and the American college of endocrinology guidelines recommend target glucose concentrations between 140 and 180 mg/dl for most general medicine and surgery patients[55]. In contrast, in more critical patients, up to 200 mg/dL might be acceptable. Insulin decreases plasma glucose and possesses a crucial anabolic activity by stimulating protein synthesis, with a consequent positive effect on muscle density and growth. It is well-known that insulin can also inhibit intracellular triglyceride (TG) lipolysis and prevent diabetic ketoacidosis. It also limits the lipotoxic effects of free fatty acids: germane to this insulin, by decreasing lipolysis, restrains ceramides' formation, which can aggravate the pro-inflammatory response [56]. Moreover, insulin can suppress the transcription of Toll-like receptors (TLRs) on circulating mononuclear cells and the expression of IL-1β and TNF released in response to infections [57]. In severe inflammatory conditions such as in COVID-19 disease, the potentially beneficial effects of intensive insulin therapy might be related to the hormone's anti-inflammatory effect beyond the antidiabetic effect. Hyperinsulinemia also antagonized the clotting system by increasing the serum levels of plasminogen activator-inhibitor-1 (PAI-1): this is important in the light of the pro-coagulant state of the patients infected by SARS-CoV-2 [58]. Since insulin is the antidiabetic agent of choice in patients with hyperglycemia and critical conditions, it is also a marker of disease severity.

**Fig. 3:**
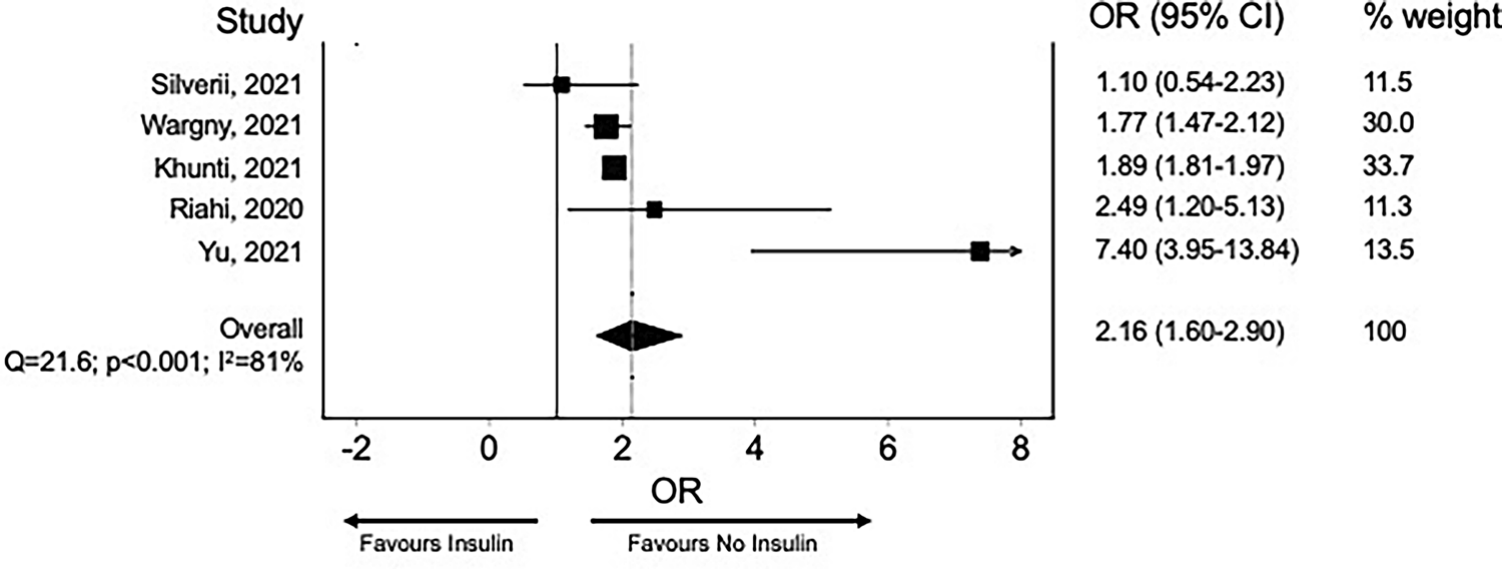
Risk for mortality for insulin users and other glucose-lowering medication users. Risk ratio with 95% confidence intervals

For this reason, insulin treatment is frequently associated with increased mortality in observational, non-randomized studies. To verify whether insulin is independently associated with increased COVID-19-related mortality we screened the English literature for studies reporting SARS-CoV-2 infection and/or COVID-19-related mortality in insulin users. As for metformin, the search string: “insulin AND COVID-19” were run in PubMed, Scopus, and Cochrane Library. The meta-analysis was performed, including all observational studies reporting mortality in patients with diabetes and COVID-19. All estimates were thus reported as risk ratios and 95% confidence interval (CI). The random-effect model was used to obtain pooled RR. Heterogeneity was assessed using the I^2^ test. Review Manager version 5.3 was used to perform the meta-analysis. Five studies were included in the analysis. As shown in Fig. 3, it can be seen that insulin treatment is associated with a more than twofold risk of mortality. However, as it can be seen, there is substantial heterogeneity among studies with an I^2^ of 86%, saying that 86% of the variability in treatment effect estimates is due to fundamental study differences and only 14% due to chance. None of the studies have performed a sensitivity analysis, so there are reasons to believe that prior insulin treatment can identify the severity of the patients' clinical conditions, and hence more prone to COVID-19 mortality. Even more problematic is that insufficient information is available on the role of in-hospital insulin treatment between those with and without prior insulin treatment. More robust data are necessary to rule out the potential anti-inflammatory effect of insulin independently of the patient’s clinical conditions and previous treatment.

## Conclusions

**Fig. 4:**
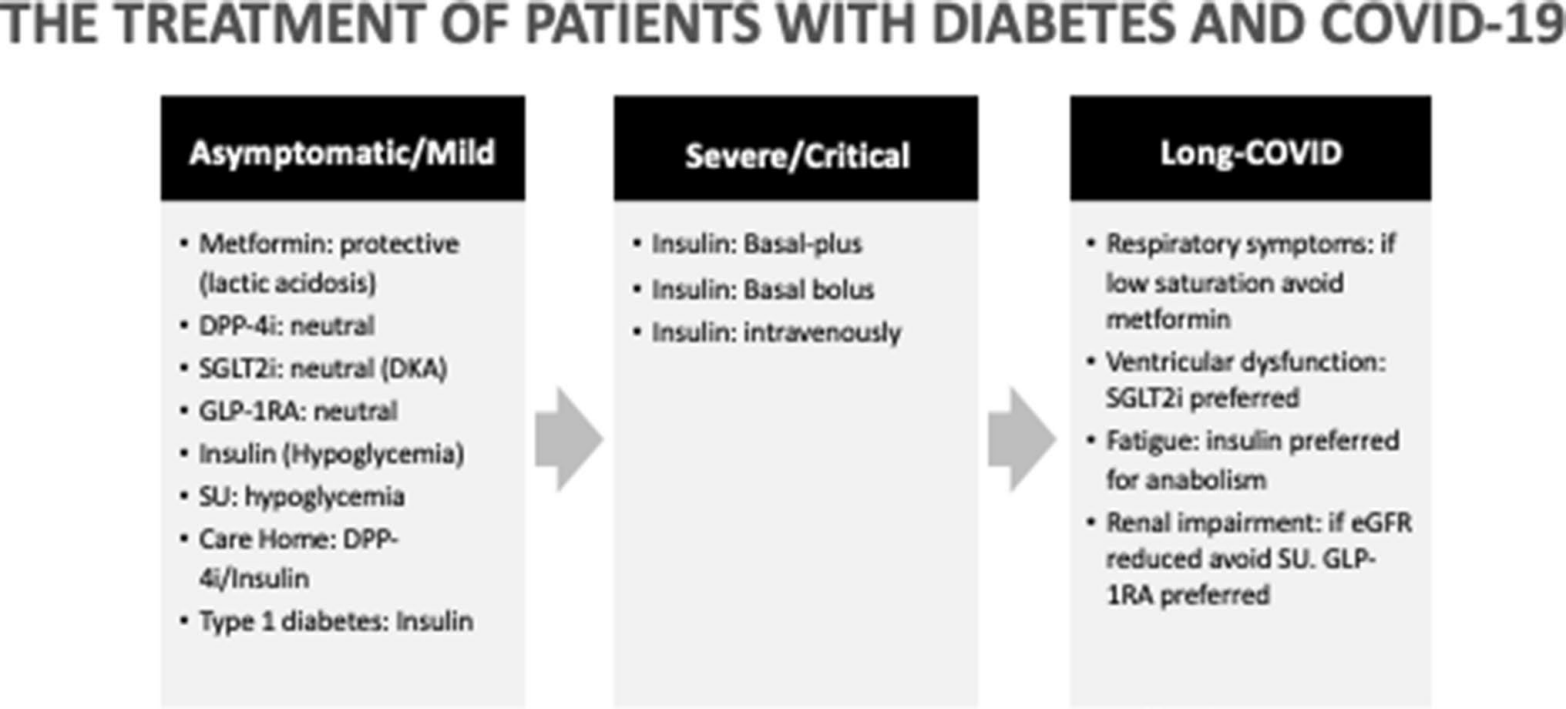
Treatment approaches to patients with diabetes and COVID-19

So: where do we start from? We reaffirm that we should start from the evidence. Overall COVID-19 infection has posed a tremendous burden to subjects with diabetes, especially for those with comorbidities and the elderly. This pandemic has also revolutionized the approach to our diabetic patients: telemedicine has facilitated the management of diabetes, although this approach needs a more widespread broadband internet and the capability to understand the use of not-easy-to-use programs [59]. Regarding the several diabetes treatment approaches, metformin appears the only one that stands for its ability to decrease death risk in those with the severe form of the disease. Yet, all available studies are retrospective, most of them are corrupted by either the lack of propensity score matching and “channeling bias” so that most of the existing results on each class of drugs are driven by the phenotype of patients likely to receive that specific drug by prescription. Another critical problem is the long-COVID [8], which can have unpredictable organ damage and outcomes, especially in patients with diabetes (Fig. 4).
